# Collapsing focal segmental sclerosis in an HIV‐negative patient

**DOI:** 10.1002/ccr3.3078

**Published:** 2020-07-03

**Authors:** Sandeep Anand Padala, Bethany Birkelo, Azeem Mohammed, Rajan Kapoor, Laura Mulloy, Prashanth Rawla

**Affiliations:** ^1^ Department of Medicine, Nephrology Medical College of Georgia Augusta University Augusta GA USA; ^2^ Department of Medicine Medical College of Georgia Augusta University Augusta GA USA; ^3^ Department of Internal Medicine Sovah Health Martinsville VA USA

**Keywords:** acute kidney injury, collapsing focal segmental glomerulosclerosis, focal segmental glomerulosclerosis, human immunodeficiency virus, obesity‐related glomerulopathy, trimethoprim‐sulfamethoxazole

## Abstract

Collapsing focal segmental glomerulosclerosis (FSGS) is classically seen in HIV‐infected patients and carries a dismal prognosis. It can also occur in HIV‐negative patients in which case, early aggressive treatment with glucocorticoids may be helpful with improvement in both proteinuria and renal function.

## Graphical abstract

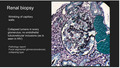

## 1 INTRODUCTION

Focal segmental glomerulosclerosis (FSGS) is a histologic lesion seen on kidney biopsy in individuals with nephrotic syndrome and is one of the most common causes of nephrotic range proteinuria and renal failure. It can be classified into primary or idiopathic and secondary FSGS. Histologically, there are five variants: Tip, Perihilar, Collapsing, Not Otherwise Specified (NOS), and Cellular. Secondary FSGS may be genetic, drug‐induced, adaptive, or due to viral infections. The collapsing variant is classically seen in human immunodeficiency virus (HIV)‐infected patients. We present a case of collapsing FSGS (cFSGS) in an HIV‐negative patient.

Nephrotic syndrome is a clinical entity constituting of heavy proteinuria (>3.5 grams/day), edema, hyperlipidemia, lipiduria, hypoalbuminemia, and loss of immunoglobulins leading to an increased risk of infections. Focal segmental glomerulosclerosis (FSGS), membranous glomerulonephritis, and minimal change disease (MCD) are the most common causes of nephrotic syndrome. Systemic diseases that cause nephrotic syndrome are diabetes mellitus, lupus nephritis, and amyloidosis. The involvement of a portion of glomeruli (focal) along with segmental sclerosis is the distinctive feature of FSGS.^1^ About 40% of all adult cases of massive proteinuria are secondary to FSGS.^2^ It can be classified into primary FSGS (idiopathic) and secondary FSGS, and the clinical presentation of both the entities is markedly different.

Patients with primary FSGS present acutely with severe proteinuria and severe hypoalbuminemia, whereas those with secondary FSGS have an indolent course with moderate proteinuria and near‐normal albumin levels. The etiology of secondary FSGS could be due to renal agenesis, viral infections, malignant hypertension, drug toxicity, obesity, renal artery stenosis, atheroembolic disease, low birth weight, reflux disease, and chronic allograft nephropathy.^2^

## 2 CASE PRESENTATION

A 25‐year‐old African American male with no known past medical history presented with a 1‐week history of intermittent headaches, constant diplopia, and dysconjugate gaze. No prior history of these symptoms was reported. The patient did not regularly see a physician. Upon admission, the vitals were BP −196/108 mm Hg, HR −89/min, RR −20/min, SpO_2_ −98% on room air, and a weight of 351 lbs (BMI 64 kg/m^2^). On fundoscopic examination, he had papilledema, but his vision was grossly normal. Cardiovascular examination revealed no murmurs and 1‐2+ edema in lower extremities. His central nervous system examination revealed a 6th nerve palsy on the left, dysconjugate gaze, facial symmetry, and midline tongue. The remainder of the examination was unremarkable. There was no personal or family history of sickle cell anemia.

**FIGURE 1:**
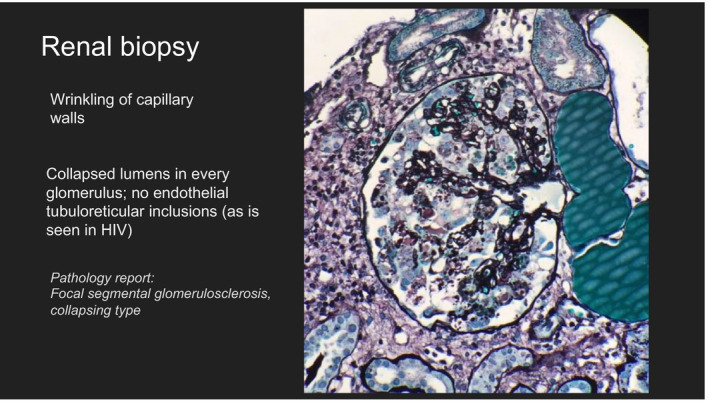
Renal biopsy showing focal segmental glomerulosclerosis, collapsing type

Laboratory data are shown in Table 1. Urinalysis was suggestive of protein >300 without any evidence of infection or blood. CT scan of the head was suggestive of partially empty sella turcica and no evidence of acute intracranial process. The patient's blood pressure improved shortly after admission. He underwent lumbar puncture (LP) which was suggestive of pseudotumor cerebri, and treatment with acetazolamide was initiated. Nephrology was consulted for an incidental finding of acute kidney injury (AKI) and proteinuria. The patient's kidney function was within normal limits based on the laboratories obtained from outside hospital during an ER visit 1 week prior to the current admission. He was diagnosed with urinary tract infection (UTI) and was prescribed Bactrim (trimethoprim and sulfamethoxazole)‐DS (double strength), one tablet twice a day for a week. Unfortunately, we were unable to obtain the results of urinalysis or any other clinical details of his ER visit, and there were no prior laboratories on our system to assess for baseline urine proteinuria. The only information we had was, he was prescribed antibiotics for UTI and the kidney functions were essentially in the normal range. The patient's history was negative for any risk factors for AKI or chronic kidney disease (CKD). Peripheral smear did not reveal any schistocytes. The spot urine protein: 369, urine creatinine: 93 (protein/creatinine ratio = 4 gm/gm). Urine microscopy did not reveal red blood cell (RBC) casts or dysmorphic RBCs. HIV antigen/antibody was negative, and the viral load was <40. Antinuclear antibody (ANA) was 1:40 speckled. Anti‐Ro, anti‐La, anti‐Smith, and anti‐dsDNA were negative. The complements C3 was within normal limits and C4 was elevated. Evaluation for infectious causes including Cytomegalovirus (CMV), Epstein Barr Virus (EBV), Parvovirus, and Hepatitis virus was all negative. C reactive protein (CRP) and erythrocyte sedimentation rate (ESR) levels were elevated at 3.243 mg/dL and 120 mm/hr respectively. Renal ultrasound revealed a 15.4 cm right kidney and 15.2 cm left kidney with increased echogenicity bilaterally without any evidence of cortical thinning or suspicious cysts, masses, or hydronephrosis. Except for the recent exposure to Bactrim, the patient was not on any other medication that could explain the cause of his AKI. Given the presentation of AKI, nephrotic range proteinuria, and negative serologic workup, a renal biopsy was obtained as shown in the image. (Figure 1). Light microscopy showed up to four glomeruli with no globally sclerotic glomeruli were identified per level. Three of the glomeruli show capillary tuft collapse with podocyte hypertrophy and numerous protein reabsorption droplets. No necrosis or crescent formation was identified. There was mild interstitial fibrosis. Many of the tubules showed dilatation with proteinaceous casts. There is a patchy, lymphoplasmacytic interstitial infiltrate. The vasculature is unremarkable. Immunofluorescence showed limited deposition of C3 within the glomeruli. Podocyte protein reabsorption droplets were seen, and ultrastructural evaluation revealed extensive podocyte foot process effacement. Electron microscopy showed hypertrophic podocytes with extensive podocyte foot process effacement without any definitive immune complex dense deposits. The glomerular basement membranes are irregularly thickened with an expansion of the lamina densa which is of unclear significance. There was no evidence of acute tubular necrosis (ATN) on the biopsy.

**Table 1:** Laboratory data

Laboratory data		
Laboratory test	Patient's value	Reference value
White blood count	10.7	4.5‐11.0 thousand/mm^3^
Red blood count	6.8	4.2‐5.5 million/mm^3^
Hemoglobin	14.6	12‐16 g/dL
Hematocrit	44.2	37%‐47%
Platelet	114	150‐400 thousand/mm^3^
Sodium	121	135‐145 mEq/L
Potassium	3.2	3.5‐5.5 mEq/L
Chloride	84	99‐109 mEq/L
Bicarbonate	27	20‐31 mEq/L
Blood urea nitrogen	58	9‐23 mg/dL
Creatinine	9.28	0.6‐1.6 mg/dL
Glucose	92	74‐106 mg/dL
Calcium	7.9	8.7‐10.4 mg/dL
Total protein	6.2	5.7‐8.2 g/dL
Albumin	3.1	3.2‐4.8 g/dL
Aspartate aminotransferase (AST)	100	0‐34 U/L
Alanine aminotransferase (ALT)	70	10‐49 U/L
Alkaline phosphatase	88	45‐129 U/L
Total bilirubin	0.3	0.3‐1.2 mg/dL
Phosphorus	6.1	2.4‐5.1 mg/dL

Treatment involved administration of 120 mg of oral Prednisone every other day, and the patient was discharged without any evidence suggesting the need to initiate dialysis as he remained nonoliguric during the entire hospital stay. Hyponatremia was corrected at an appropriate rate. Subsequent nephrology clinic visits revealed improving renal indices. One week after the discharge, patient's laboratories revealed BUN of 55 mg/dL with a Cr of 4.52 mg/dL. Three months after discharge, his BUN was 17 mg/dL with a creatinine of 1.67 mg/dL at which point steroid taper was initiated. The spot urine protein: 769 and spot urine creatinine: 321.5 (protein/creatinine ratio = 2.39 gm/gm). 24‐hour urine collection was not performed.

## 3 DISCUSSION

Histologically, FSGS is classified into five variants, including Cellular, Tip, Collapsing, Not Otherwise Specified (NOS) or Classical, and Perihilar.^3^ Obesity‐related glomerulopathy (ORG) is a subset of secondary FSGS that occurs in morbidly obese patients with a body mass index (BMI) of more than 30 kg/m^2^, without clinical and histologic evidence of other renal diseases.^4^, ^5^, ^6^, ^7^

Across the globe, cFSGS is an aggressive form of FSGS leading to ESRD.^7^, ^8^, ^9^, ^10^, ^11^ In the early 1970s, there were case reports describing similar clinical and pathological features as malignant FSGS.^9^ In 1986, Weiss et al described a distinct clinical variant in six young African American populations.^12^ Eventually in the 1990s, Detwiler et al and Valeri et al described case reports with similar lesions as an FSGS variant^13^, ^14^; subsequently, the lesion was considered to be a variant of FSGS and classified as cFSGS by the Columbia classification of FSGS.^15^ More recently, cFSGS has been diagnosed in biopsies of native and transplant kidneys all across the world, suggesting prior diagnostic bias.^16^, ^17^ According to the podocytopathies classification, it has been recently proposed that cFSGS be classified separately from FSGS. In an experimental model of acutely induced proteinuria, Agarwal V et al observed wide‐spread effacement of foot processes before the onset of proteinuria.^18^ In a retrospective review of 93 patients by Jeroen et al, the frequency of NOS was 32%, 37% tip, 26% perihilar, and 5% collapsing. NOS and perihilar groups had less frequency of nephrotic syndrome in comparison with the tip variant group; however, tip variant had better baseline creatinine.^19^, ^20^ Glomerular sclerosis and hyalinosis was lowest in the tip variant, moderate in the NOS and most severe in patients with perihilar variant. Renal survival was better at 5 years with the tip variant. The serum creatinine concentration and type of FSGS were significant independent predictors of renal survival.^20^

Focal segmental glomerulosclerosis and MCD represent the same spectrum of disease affecting the podocytes, and the extent of foot process effacement differentiates them. The degree of effacement in MCD is diffuse, while FSGS can have variable degrees of effacement. Compared to secondary FSGS, the degree of effacement is more in primary FSGS. Jeroen et al also studied the foot process effacement in FSGS compared to MCD and the normal kidneys, and demonstrated that the foot processes are more effaced in idiopathic FSGS NOS and tip variant than in MCD.^21^ These findings suggest that some forms of idiopathic FSGS and MCD may have a single underlying cause. In selected cases, the measurement of FPW can be useful to distinguish between idiopathic FSGS and FSGS secondary to maladaptive responses.^21^ Van den Berg JG et al confirmed the previous findings proposing that the nature of the underlying disease is the main dependent factor for the degree of foot process effacement.^22^ Using a multivariate analysis, the results indicated that the type of disease was the only determinant of FPW; this correlates with more recent insights into podocyte biology, indicating that both proteinuria and morphological alterations in podocytes or slit pores are consequences of podocyte injury.^21^, ^23^, ^24^, ^25^ In addition, there is evidence of podocyte injury secondary to circulating factors in idiopathic FSGS and MCD.^26^, ^27^, ^28^ Thus far the circulating factor has not been identified. The difference in foot process effacement in idiopathic FSGS and MCD in our case suggests that the underlying cause of podocyte injury differs between the two disorders.^21^ This is in agreement with the notion that different plasma factors appear to be involved in idiopathic FSGS and MCNS.^21^, ^29^ About 80% of the cases are primary or idiopathic FSGS. Among all, NOS and Tip variants are more common. cFSGS is strongly associated with HIV infection. Careful attention has to be paid to the biopsy, as the findings of cFSGS can mimic the Cellular FSGS and Crescentic glomerulonephritis.

Clinically cFSGS patients exhibit a higher degree of renal insufficiency, proteinuria, and progression to ESRD when compared to other variants of FSGS. There are no prospective trials for the treatment of cFSGS. The recommended regimen for the treatment of FSGS consists of high dose oral prednisone: 1 mg/kg/day, 80 mg/day, or 120 mg every other day with a slow taper over 6 months. High dose steroids can be used for a minimum of 4 weeks and a maximum of 16 weeks as tolerated or until complete remission is achieved. Immunosuppressive therapy with cyclosporine or mycophenolate mofetil should be considered when there is no response to the steroids.^30^ Other medications include ACE inhibitors and statins as tolerated. Complete remission occurs in less than 10% of patients and a partial response in 15.2% of patients.^9^ In most series, the incidence of ESRD has been reported in 50%‐100%.^7^, ^8^, ^9^, ^10^, ^11^ Valeri et al revealed that ESRD may be predicted by serum creatinine at the time of biopsy and lack of remission of proteinuria.^9^ Laurinavicius et al reported that the risk of ESRD was increased with a serum creatinine of more than 2 mg/dL, proteinuria >8 g/day, HIV infection, more than 20% of interstitial fibrosis and collapsing lesions.^11^

Collapsing FSGS generally carries the worst prognosis of all the variants; however, if remission is achieved, patients have similar outcomes as the other variants. Recently, immediate LDL‐Apheresis (low‐density lipoprotein) therapy was reported to be effective for drug‐resistant nephrotic syndrome, particularly FSGS.^31^, ^32^ Persistent LDL elevation can induce glomerular and tubulointerstitial damage. The bioavailability of steroid and calcineurin inhibitors is impaired in hyperlipidemic conditions, which reinforces the belief that lowering the lipids by LDL‐A is effective.^32^ A multicenter prospective study, POLARIS (Prospective Observational Survey on the Long‐Term Effects of LDL Apheresis on Drug‐Resistant Nephrotic Syndrome), revealed that LDL Apheresis might provide early remission in approximately half of the drug‐resistant nephrotic syndrome patients (25/47, 53.1%).^32^, ^33^

Apart from HIV infection, the other conditions associated with cFSGS include infection (CMV, EBV, parvovirus B19, tuberculosis, malaria, and leishmaniasis), autoimmune disease (SLE, adult still's disease, and connective tissue disorder), glomerular (thrombotic microangiopathy, athero‐embolism, renal infarction, superimposed on IgA and diabetic nephropathy), malignancy (hemophagocytic lymphohistiocytosis, multiple myeloma, and leukemia), drugs (bisphosphonates, interferon, anabolic steroids, calcineurin inhibitors, and mTOR inhibitors), and post‐transplantation.^9^ A study by Kambham et al showed that the biopsy incidence of ORG has gone up by 10‐fold and nearly half of the patients had nephrotic range proteinuria with mean 24‐hour protein lower than idiopathic FSGS.^4^ The pathophysiology of ORG is multifactorial, which includes elevated renal plasma flow and glomerular filtration rate,^34^ glomerular hypertrophy due to hyperinsulinemia mediated insulin growth factors(IGF 1and IGF2),^35^ increased glomerulosclerosis due to transforming growth factor (TGF) due to elevated leptin levels ^36^ and hyperlipidemia induced podocyte toxicity.^37^

## 4 CONCLUSION

The patient had idiopathic collapsing FSGS. The workup for other conditions associated with collapsing FSGS was unrevealing. The review of the literature provided no mention of pseudotumor cerebri as an associated condition. Given the patient's BMI of 64, there was initial consideration of an obesity‐related glomerulopathy; however, the acuity of clinical presentation, degree of renal insufficiency, and biopsy findings did not support this. The recent exposure to TMP‐SMX (trimethoprim and sulfamethoxazole) may have contributed to the patient's renal insufficiency; however, the bland urine sediment and absence of significant interstitial infiltrate viewed on the renal biopsy do not support a diagnosis of acute interstitial nephritis. While TMP‐SMX is also known to cause AKI by mechanisms other than AIN, studies of AKI associated with trimethoprim show a significantly lesser degree of creatinine elevation. While collapsing FSGS generally carries the most dismal prognosis of all the FSGS variants, this case provides evidence that early aggressive treatment with glucocorticoids may be helpful, with improvement in both proteinuria and renal function. Additionally, this case illustrates the importance of prompt nephrology consultation and consideration of an early renal biopsy in a patient with AKI and nephrotic range proteinuria, as the biopsy findings directly impacted the management of care in this young man.

## CONFLICT OF INTEREST

None declared.

## AUTHOR CONTRIBUTIONS

SP and BB: have made substantial contributions to conception and design, or acquisition of data, or analysis and interpretation of data. SP, BB, AM, RK, LM and PR: been involved in drafting the manuscript or revising it critically for important intellectual content. SP, BB, AM, RK, LM and PR: given final approval of the version to be published. Each author should have participated sufficiently in the work to take public responsibility for appropriate portions of the content. SP, BB, AM, RK, LM and PR: agreed to be accountable for all aspects of the work in ensuring that questions related to the accuracy or integrity of any part of the work are appropriately investigated and resolved.
